# Retraction: Impaired potentiation of theta oscillations during a visual cortical plasticity paradigm in individuals with schizophrenia

**DOI:** 10.3389/fpsyt.2026.1868461

**Published:** 2026-05-06

**Authors:** Frontiers Editorial Office

The authors retract the 16 December 2020 article cited above.

Following publication, the authors contacted the Editorial Office to request the retraction of the cited article, stating that they recently reviewed the raw EEG data from this study and discovered data file errors affecting the data of participants in the HC group, and that they were subsequently unable to recover the original data files. An investigation was conducted in accordance with Frontiers’ policies that confirmed this; therefore, the article has been retracted.

The authors are revising their paper using the intact subgroup of patients to be considered by the journal as a new submission.

This retraction was approved by the Chief Editors of Frontiers in Psychiatry and the Chief Executive Editor of Frontiers. The authors agree to this retraction.

